# Adhesive Tissue Engineered Scaffolds: Mechanisms and Applications

**DOI:** 10.3389/fbioe.2021.683079

**Published:** 2021-07-20

**Authors:** Shuai Chen, Carmen J. Gil, Liqun Ning, Linqi Jin, Lilanni Perez, Gabriella Kabboul, Martin L. Tomov, Vahid Serpooshan

**Affiliations:** ^1^Department of Biomedical Engineering, Emory University School of Medicine, Georgia Institute of Technology, Atlanta, GA, United States; ^2^Department of Pediatrics, Emory University School of Medicine, Atlanta, GA, United States; ^3^Children’s Healthcare of Atlanta, Atlanta, GA, United States

**Keywords:** adhesive tissue engineering scaffold, tissue regeneration, scaffold, bone regeneration, cartilage regeneration, nerve regeneration, cardiac regeneration, wound repair

## Abstract

A variety of suture and bioglue techniques are conventionally used to secure engineered scaffold systems onto the target tissues. These techniques, however, confront several obstacles including secondary damages, cytotoxicity, insufficient adhesion strength, improper degradation rate, and possible allergic reactions. Adhesive tissue engineering scaffolds (ATESs) can circumvent these limitations by introducing their intrinsic tissue adhesion ability. This article highlights the significance of ATESs, reviews their key characteristics and requirements, and explores various mechanisms of action to secure the scaffold onto the tissue. We discuss the current applications of advanced ATES products in various fields of tissue engineering, together with some of the key challenges for each specific field. Strategies for qualitative and quantitative assessment of adhesive properties of scaffolds are presented. Furthermore, we highlight the future prospective in the development of advanced ATES systems for regenerative medicine therapies.

## Introduction

Traditionally, adhesive biomaterials are classified into hemostats, sealants, and tissue adhesives (Lauto et al., 2008). Hemostats mainly function by increasing blood coagulation (Hickman et al., 2018). Sealants are the ones that adhere to tissues and act as a barrier to prevent leakage (Sanders and Nagatomi, 2014). Meanwhile tissue adhesives provide stronger adhesive ability to hold tissues together (Burks and Spotnitz, 2014; Ge and Chen, 2020). Conventional tissue adhesives and sealants could be used in cases of blood vessel anastomosis, lung leakage preventions, and incision closure. Examples of tissue adhesives and sealants are cyanoacrylates, albumin, glutaraldehyde, polyethylene glycol (PEG) polymers, and fibrin sealant (Ge and Chen, 2020; Nam and Mooney, 2021). Tissue adhesives and sealants are also used as glue for the application of non-adhesive scaffold devices, aiding to fix the scaffold on the surface of organs and tissues (Ma et al., 2021). However, most tissue adhesives and sealants lack the specific requirements for use as a proper scaffold system for tissue regeneration. Main limitations include: (1) tissue adhesives and sealants are typically used to close incisions but not qualified for filling in larger gaps and defects (Shirzaei Sani et al., 2019); and (2) tissue adhesives and sealants, although showing a degree of biocompatibility and biodegradability, are not specifically designed to support various cellular activities that are needed for tissue regeneration and usually cause side effects. For example, fibrin sealants may cause viral or infection complications. They also usually lack enough adhesive strength (Spotnitz, 2014). Cyanoacrylate could cause inflammation by toxic degradation products and exothermic reaction by polymerization (Pascual et al., 2016). Also, its stiffness may not be compatible to soft tissues. Albumin and glutaraldehyde have side effects such as infection and delayed wound healing (Furst and Banerjee, 2005). PEG lacks proper biodegradability and may have a chronic inflammation response and potential of swelling up to 350 to 400% of its volume (Lauto et al., 2008; Burks and Spotnitz, 2014; Bhagat and Becker, 2017; Malki et al., 2018). These side effects disqualify most of these materials as proper cell carriers and ECM analogs, and prevent their usage in large quantities when applied to human body.

To address the limitations of traditional adhesive biomaterials, adhesive tissue engineering scaffolds (ATESs) have been developed to repair damaged tissues and guide tissue regeneration after trauma and degeneration (Vermonden et al., 2008; Wiltsey et al., 2015; Ark et al., 2016). As a new generation of adhesive systems, ATESs provide a 3-dimensional (3D) biomimetic and highly biocompatible environment for cell adhesion, growth, differentiation, proliferation, secretion of extracellular matrix (ECM) proteins, as well as remodeling and replacement of the scaffold with regenerated tissue during matrix degradation (Ark et al., 2016; Boyadzhieva et al., 2019). Notably, ATESs can firmly adhere onto the tissue surface without the help of glue, sutures, or other additional fixtures, while providing the desired functions of the scaffolds (Boyadzhieva et al., 2019). ATESs could offer the following benefits: (1) they can be delivered and secured onto narrow or complicated structures in the human body where suturing or gluing might be difficult or impractical (Salzlechner et al., 2020); (2) secondary damages by suturing and bio-incompatibility of commercial glues, such as toxicity of cyanoacrylate or allergies caused by fibrin glues, can be avoided; (3) the delivery of ATESs could be achieved through conduits or syringes, avoiding highly invasive operations; (4) the hindered cell migration between tissues and scaffolds caused by glue or other fixtures with low biocompatibility can be circumvented (Shin et al., 2019); and (5) specific scaffold systems, such as microgel sphere assemblies, can be readily integrated with the surrounding ECM (Xin et al., 2018). Therefore, by combining the advantages of functional scaffolding systems for cell growth and tissue regeneration, and the benefits of intrinsic adhesive products, ATESs can facilitate surgical operations and provide safer medical treatments for patients.

Over the past decade, ATESs have found increasing applications in the repair and regeneration of various organs and tissues, such as cartilage, bone, ocular, nerve, heart, and skin. Adhesive scaffolds can be engineered using different types of biomaterials, including hydrogels, assembled microgel spheres, foams, and electrospun patches. Despite the rapid advancement of the field, there are only a small number of review articles on the ATES systems. For instance, Hozumi and Nomizu reviewed the current progress made on the peptide-conjugated chitosan hydrogel systems as targeted cell-adhesive scaffolds in tissue engineering (Hozumi and Nomizu, 2018). The article mainly focused on the peptide–chitosan matrices and their applications for analyzing cell-biomaterial interactions. Thi et al. published a review on horseradish peroxidase (HRP)-catalyzed hydrogel as adhesive materials. However, the review focuses on the use of HRP-catalyzed hydrogels for hemostasis and drug and cell delivery purposes (Thi et al., 2019). Pei et al. also published a review on the polymer hydrogel bioadhesives, with a small section about bioadhesives for tissue engineering applications (Pei et al., 2021). In this article, we aim to provide a comprehensive review on a variety of tissue engineering scaffolds with adhesive properties. We will elaborate the specific requirements of ATES systems, their adhesion mechanisms, and applications in tissue engineering and regenerative medicine.

## Main Characteristics and Requirements of Atess

### Basic Requirements of ATESs

In general, ATESs are designed to serve two purposes: adhesion (fixation) onto the tissue surfaces and mimicking the ECM niche for cell proliferation, differentiation, growth, to restore tissue structure and function. Based on these primary functions, the following properties are required for a 3D scaffold system to qualify as an ATES (Table 1): (1) sufficient adhesive properties to tolerate wet and dynamic *in vivo* environment and the various forces that exist; (2) biocompatibility and low cell toxicity that enable cell survival and function, as well as integration with the surrounding (host) native tissue; (3) proper biodegradation and swelling behavior that accommodates the tissue regeneration rate; (4) incorporated porosity and vasculature that provide sufficient oxygen and nutrients; (5) Young’s modulus and stiffness that resemble those of the native tissue; and (6) elasticity or flexibility to withstand tensional or dynamic forces in cases such as nerve or myocardial regeneration (Lauto et al., 2008; Zaokari et al., 2020).

**TABLE 1:** Main required properties for adhesive tissue engineering scaffolds (ATESs).

**Property**	**Characterization method**	**Design considerations**	**Approach considerations**	**Target value**
Adhesive properties	Tensile adhesion test; shear adhesion test; wound closure test; burst pressure test; peeling test	Adhesion firmly after applying and in long term; tolerance of wet condition and stresses	Implying covalent and non-covalent interactions	Adhesion strength 1KPa–1MPa
Biocompatibility and low cell toxicity	AlamarBlue; MTT; *in vivo* compatibility tests	Low cell and tissue toxicity that allow cell growth and tissue regeneration	Using bio-compatible materials and adhesion mechanisms	Usually higher cell survival rates are preferred.
Biodegradation and swelling behavior	*In vivo* and *in vitro* degradation and swelling tests	Low swelling ratios that do not affect design pattern or exert pressure to tissue; proper degradation behavior that accommodates tissue regeneration rate	Choosing proper materials with intrinsic low swelling behavior and proper degradation rate; proper crosslink density; proper chain length for polymers	Low swelling ratio is preferred; 20–25% of materials is left after 4 weeks of degradation *in vivo*
Porosity and vasculature	SEM; microscopy	Incorporation of vasculature or choosing materials with adequate porosity	3D printed vascular system or choosing a proper base material and proper concentration and crosslink density	Optimal porosity and pore size highly depend on the tissue type and the specific application
Young’s modulus and stiffness	Mechanical tests: indentation test; compression test	Strong enough for bone and cartilage repair; soft enough for patient comfort for corneal repair; ability to withstand tensile stress for nerve repair	Choosing proper material, concentration, and crosslink density	1 KPa–100 MPa for cartilage and bone; 100 Pa – 100 KPa for corneal; and typically 100 Pa – 100 KPa for other tissues

### Adhesion Mechanisms for ATESs

**FIGURE 1:**
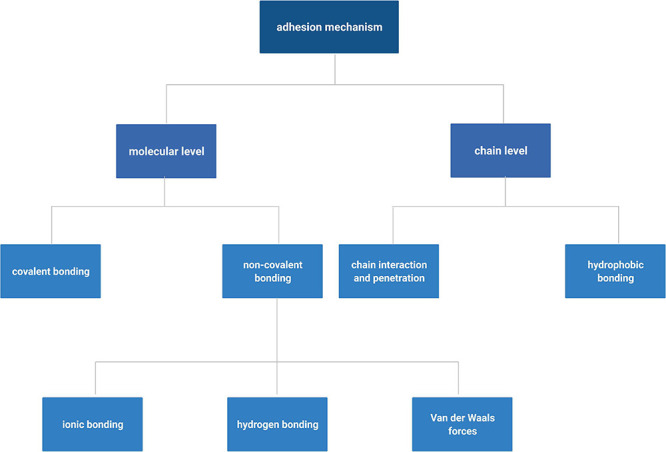
Summary of different mechanisms of adhesion of tissue engineering scaffolds. Created with BioRender.com.

Adhesion to ATES requires interaction between surfaces of the scaffold and the recipient tissue, which could be achieved by molecular interactions and chain penetration and entanglement (Figure 1). Generally, binding in the molecular level between the scaffold and tissue can be categorized into ionic, covalent, hydrogen, Van der Waals, and hydrophobic bonding (Korde and Kandasubramanian, 2018). Ionic bonding is based on electrostatic interactions between positive charges of scaffold polymers, such as chitosan, and negative charges on cell surfaces (Gåserød et al., 1998). Covalent bonding, achieved by forming strong bonds through sharing electrons in pairs, is a commonly used strategy to achieve tough and persistent adhesive properties. Functional groups, such as succinimidyl succinate or catechol groups that chemically react with amine moieties on the tissue surface, can be introduced to the back bone of scaffold polymers and anchor the construct to the target tissue (Simson et al., 2013; Han et al., 2017). Hydrogen bonding is weaker than the ionic or covalent bonding, however, it offers the ability to reform after deformation in contrast to most covalent bonds. The hydrogen bond is the driving force of supramolecular adhesives and can also be used as a supplemental force when scaffold material is protein or polysaccharide based. Van der Waals bond is even weaker than hydrogen bonding and provides supplementary force for tissue adhesion. Hydrophobic bonding is the entropy induced molecular interaction and aggregation within non-polar molecules under aqueous environment. Hydrophobic domains on scaffolds could interact with fibronectin and fibrillin in the ECM, on the surface of tissues, and improve adhesive strength (Nishiguchi et al., 2019; Nishiguchi and Taguchi, 2020). Specifically, such effect can improve adhesive properties under wet conditions by decreasing water layer at the interface between the scaffold and host tissue through repelling water molecules, which helps addressing one of the most challenging issues for adhesion *in vivo* (Wang B. et al., 2018). In a more recent effort, an adhesive hydrogel, consisting of polyacrylic acid, chitosan, tannic acid, and Al^3+^, demonstrated strong and reversible underwater adhesion properties, owing to its electrostatic interactions and dynamic catechol chemistry (Duarte et al., 2020).

In addition to introducing functional groups, positive charges, and hydrophobic domains, increasing chain penetration into tissue is another way to achieve higher adhesive efficiency. One example is incorporating free PEG polymers that can increase adhesive potentials through free chain interpenetration into mucosa surfaces (Huang et al., 2000). It should be noted that in the design of ATESs, the introduction of adhesive properties should not affect biocompatibility, cell affinity, porosity, and biodegradation characteristics of the scaffold system for the optimal regenerative effects.

When applied *in vivo*, the interaction of the scaffold with body fluids and blood could affect its adhesive properties. Water molecules can form a boundary between the adhesive scaffold and tissue, mask the functional groups, and thus hinder adhesion processes. Non-covalent interactions can also be affected (Hou et al., 2020a). Efforts have been made to improve underwater adhesive properties through mimicking the mechanism that are active in marine animals such as mussels and sandcastle worms (Zhao et al., 2017). The keys to achieve underwater adhesion ability are the incorporation of L-3,4-dihydroxyphenylalanine (Dopa) and complex coacervation. Dopa provides a reversible chelation as well as covalent bonding with thiols and amines after oxidation. In complex coacervation, a denser liquid phase, separated from two fluid phases containing oppositely charged polyelectrolytes, binds to the wetted surface and triggers the underwater adhesion (Oh et al., 2014). In addition to these key mechanisms, other methods to enhance adhesion *in vivo* include hydrophobic interaction, double layer adhesion by zwitterions, increased surface unevenness of hydrogels (improves contact with adherends by repellence of liquid), incorporation of polymers that interpenetrate into the adherend and form interactions with chains of the substrate, and water absorbable dehydrated gelatin and poly (acrylic acid) films (Laura et al., 2017; Nishiguchi et al., 2019; Yuk et al., 2019; Hou et al., 2020a). In the case of ATESs, compatibility with native cells and tissues and the feasibility to form a 3D shaped construct should be also considered when selecting optimal methods for *in vivo* adhesion ability under wet conditions.

Adhesion under dynamic forces has been also a challenging concept. Adhesive cardiac scaffolds [i.e., cardiac patches (Serpooshan et al., 2013b, 2014; Serpooshan and Ruiz-Lozano, 2014)] applied to the surface of a beating heart are an example (Lin et al., 2019; Walker et al., 2019). The irregular and dynamic shape of the heart complicates maintaining the patch biomaterial in place, before curing and bonding formation with the tissue (Walker et al., 2019). The slippery wet surface together with the pulsatile motion make the cardiac patch adhesion one of the most challenging tasks, which requires strong adhesion and short curing and adhesion times under wet conditions. In the next section we will discuss advanced material options that been developed to meet such requirements.

Other mechanical requirements for ATES devices include tolerance to tension in applications such as nerve repair (Du et al., 2018), and to compression in cartilage and bone regeneration (Serpooshan et al., 2010, 2013a). Further, flexibility and elasticity of the scaffold are prominent in applications for lung and gastrointestinal adhesion (Tsuchiya et al., 2020). Also, harsh conditions in diseased states or post-surgery should be taken into account for *in vivo* applications (Zaokari et al., 2020). Such harsh conditions, such as low pH, oxidative environment, and high immune response, can affect degradation rate, swelling ratio, and cohesive strength of scaffolds, leading to a diminished adhesive strength in the long term (Taboada et al., 2020). In particular, lower pH can block amines on the tissue surface by amine protonation and hinder scaffold adhesive behavior based on covalent bonding with amine groups (Taboada et al., 2020). In summary, to manufacture an optimal ATES device for clinical and translational applications, the following material requirements must be fulfilled: (1) strong and durable adhesive properties; (2) the ability to form adhesion under wet conditions; (3) sufficient adhesive properties after partial degradation and swelling; (4) tolerance to tensile, compressive, and dynamic forces; and (5) sufficient adhesive strength under inflammatory conditions.

### Measurement of Adhesion Properties of ATESs

**FIGURE 2:**
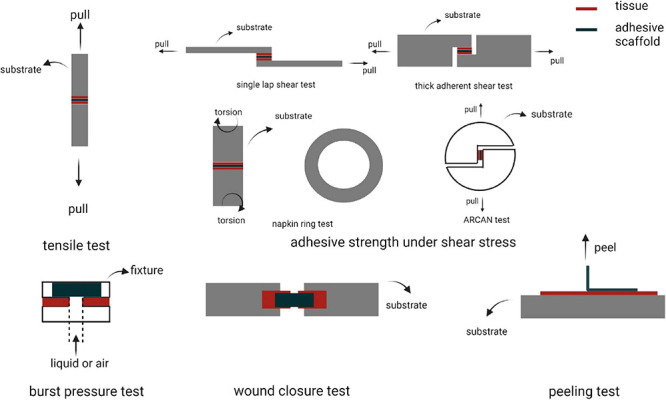
Different methods to assess adhesion strength. Created with BioRender.com.

Adhesive strength is the core property of the ATESs. A variety of mechanical tests such as tensile strength, shearing strength, burst pressure, wound closure, and peeling adhesion tests are primarily used to probe adhesive strength of biomaterials (Figure 2; Shin et al., 2015). Tensile test is employed when the adhesive scaffold is used to provide a linkage, such as in nerve repairing implants (Muzhou et al., 2012; Assmann et al., 2017; Xin et al., 2017; Jouan and Constantinescu, 2018; Chandrasekharan et al., 2019; Hong et al., 2019; Yuk et al., 2019; Cadena et al., 2020). Tensile test to measure adhesive properties is conducted by attaching the scaffold between the target tissues that are connected to the two probes of a tensile tester. After sufficient time for adhesion, the probe is pulled at a determined speed and the tensile strength is recorded (Shin et al., 2015). For shearing adhesive strength, different setups are used based on variable geometries and ways of applying the shearing forces. A single lap shear test, thick adherent shear test, and the Arcan device (butterfly shape) transform tensile movement to shear forces, while napkin ring test uses a torsion loading mode (Muzhou et al., 2012; Jouan and Constantinescu, 2018). Burst pressure test investigates the capability of scaffold to withstand air or fluid pressure. The tissue is fixed onto a device linked to a syringe pump. Subsequently, an incision of certain size is made in the tissue. After application of adhesive material to the damaged region and certain time for adhesive curing, liquid or air is applied with increasing pressure to the sample until bursting. The burst pressure is recorded as the highest pressure that the adhesive material could withstand before breakage (Assmann et al., 2017; Hong et al., 2019). Wound closure test can be examined according to ASTM F2458-05 standard. Two ends of a tissue piece for testing are glued to two slides and left with a gap. After incision in the middle, the tissue is re-united with adhesive material and then pulled by tensile stress until detachment (Chandrasekharan et al., 2019). Peeling test is used to measure interfacial adhesion toughness and is measured from the plateau force for either 180-degree or 90-degree peel test (Xin et al., 2017; Yuk et al., 2019).

Qualitative (or semi-quantitative) methods such as lifting heavy objects by adhesive material or twisting or bending the bound materials are also frequently used to demonstrate adequate adhesive properties (Liu X. et al., 2017). To better mimic the *in vivo* environment, tests could be conducted in aqueous solutions or by pre-wetting tissue scaffolds (Shoo and Stewart, 2010). Humid chambers could also be used to maintain moisture content and prevent dehydration (Mehdizadeh et al., 2012). Considering the complex geometries of tissues and the diverse loading types that can be applied to the adhesion site, a combination of multiple adhesion tests may be a better strategy for comprehensive analysis of the binding strength. Other properties such as swelling ratio, mechanical properties, biodegradability, porosity, and biocompatibility are extensively examined for ATES systems (Zhou et al., 2016; Han et al., 2018).

## Applications of Atess

Due to many unique advantages that discussed above, ATES systems are being increasingly used in a variety of tissue engineering applications (Table 2). Here we review six of the most common fields of tissue engineering that have utilized adhesive scaffolds. For each case, we discuss the necessity of regenerative treatments, the challenges that current therapies face in each field, and the significance and the outlook of applying ATES devices as an alternative approach. Benefits of these adhesive scaffold systems in comparison to conventional tissue engineering strategies are elaborated.

**TABLE 2:** List of various adhesive tissue engineering scaffold (ATES) systems along with the scaffold type, adhesion mechanism, and applications.

**Organ, Tissue**	**Scaffold type**	**Adhesion mechanism**	**Material(s)**	**Application(s)**	
Nerve	Hydrogel	Covalent bonding (reaction between methacrylates and amines)	GelMA and MeTro	*In vitro* support of Schwann cell growth, outgrowth of encapsulated dorsal root ganglia	(Soucy et al., 2018)
		Hydrogen bonds, π-cation, and electrostatic interactions	Chitosan and catechol modified ε-polylysine	*In vivo* repair of transected nerve fiber	(Zhou et al., 2016)
Cartilage	Hydrogel	Covalent bonding (Schiff’s reaction)	Gelatin, borax, and oxidized alginate	*In vitro* support of chondrocyte proliferation and migration	(Balakrishnan et al., 2014)
		Covalent bonding (conjugation of tyramines and tyrosines)	Sulfate and tyramine modified alginate	*In vitro* support of viability and re-differentiation of chondrocytes, *in vivo* support of secretion of chondrocytes	(Ztürk et al., 2020)
		Covalent bonding (reaction between methacrylates and amines)	elastin-like polypeptide (ELP) combined with methacrylate modified hyaluronic acid (MeHA)	*In vitro* support of proliferation and migration of hMSCs and NIH-3T3 cells	(Shirzaei Sani et al., 2018)
		Covalent bonding (reaction between quinone groups and amine, imidazole, and thiol groups)	Gelatin and tyramine modified hyaluronic acid	*In vitro* support of viability, proliferation, and promotion of rabbit meniscus fibro-chondrocytes	(Kim et al., 2018)
		Covalent bonding (Schiff’s reaction) for PNIPAAm-g-CS combined with aldehyde-modified chondroitin sulfate; hydrogen bonding and ionic bonding for PNIPAAm-g-CS with calcium alginate particles	Chondroitin sulfate grafted poly(N-isopropylacrylamide) (PNIPAAm-g-CS) combined with aldehyde-modified chondroitin sulfate; or PNIPAAm-g-CS with calcium alginate particles	*In vitro* support of viability of adipose derived stem cells and HEK-293 cells	(Wiltsey et al., 2015)
		Covalent bonding (reaction between quinone groups and amino groups)	Catechol containing poly(2-alkyl-2-oxazoline) based polymers and fibrinogen	*In vitro* support of chondrocyte penetration, secretion, and cartilage tissue regeneration	(Berberich et al., 2019)
		Hydrogen bonds, π-cation and electrostatic interactions	Polydopamine-chondroitin complex and polyacrylamide	*In vitro* support of proliferation and gene expression of bone marrow stem cells and chondrocytes; *in vivo* cartilage repair	(Han et al., 2018)
		Covalent bonding (reaction between quinones and amino groups and between methacrylates and amines)	Methacrylate and 3,4-dihydroxyphenylalanine modified hyaluronic acid	*In vitro* adhesion to mouse hind limbs and support of 17IA4 cell viability	(Salzlechner et al., 2020)
	Micro-particles	Covalent bonding (Schiff’s reaction)	N-(2-aminoethyl)-4-(4-(hydroxymethyl)-2-methoxy-5-nitrosophenoxy) butanamide decorated silk fibroin microparticles	*In vivo* cartilage regeneration	(Zhang et al., 2020)
		Covalent bonding (reaction between PEG-NHS and amines)	norbornene-modified gelatin crosslinked by thiol-modified PEG	*In vitro* support of viability and secretion of hBMSCs. and cartilage tissue regeneration	(Fanyi et al., 2018)
Cornea	Hydrogel	Covalent bonding (reaction between methacrylates and amines)	GelMA	*In vivo* repair of stromal and re-epithelialization of corneal defects	(Shirzaei Sani et al., 2019)
		Hydrogen bonds, π-cation and electrostatic interactions	Dopamine modified hyaluronic acid	*In vitro* support of viability and expression of hASCs and LESC	(Koivusalo et al., 2019)
Skin	Hydrogel	Covalent bonding (reaction between methacrylates and amines)	GelMA	*In vivo* repair of wounds and promotion of vascularization	(Saleh et al., 2019)
		Covalent bonding (amide bonds)	N-hydroxysuccinimide modified chondroitin sulfate cross-linked by PEG–(NH_2_)_6_	*In vitro* support of viability of chondrocytes	(Strehin et al., 2010)
		Hydrogen bonds and electrostatic interactions	polyurethane-poly(acrylamide)	*In vivo* repair of wounds	(Hou et al., 2020b)
		Non-covalent hydrogen bonding generated between urethane esters and tissues	poly(ethylene glycol)and poly(sulfamethazine ester urethane) copolymer	*In vivo* repair of wounds	(Duy et al., 2018)
		Hydrogen bonds and ionic interactions	Gelatin connected PCLA-bPEG-b-PCLA	*In vivo* repair of wounds	(Turabee et al., 2019)
Heart	Hydrogel	Covalent bonding (reaction between quinone groups and amino groups)	Catechol modified hyaluronic acid	*In vivo* treatment of myocardial infarction	(Shin et al., 2019)
	Electro-spun Patch	Covalent bonding (reaction between methacrylates and amines) and ionic bonds	choline-based bio-ionic liquid conjugated Gel MA	*In vivo* treatment of myocardial infarction	(Walker et al., 2019)
		Denatured protein interlock	Albumin	*In vivo* adhesion to the heart surface	(Malki et al., 2018)
Bone	Hydrogel	Covalent bonding (reaction between methacrylates and amines), together with hydrogen bonds, π-cation and electrostatic interactions	Dopamine modified methacrylated alginate	*In vivo* bone regeneration	(Hasani-Sadrabadi et al., 2020)
		Covalent bonding (Schiff’s reaction)	Aldehyde modified hyaluronic acid	*In vitro* proliferation of hMSC	(Bermejo-Velasco et al., 2019)
		Non-covalent nucleophile-phenolic bonding and Ca^2+^-phenolic coordination bonds	Hydroxyapatite, tannic acid and silk fibroin	*In vitro* growth and differentiation of rat bone MSCs and *in vivo* repair of bone	(Bai et al., 2020)
	Foam	Non-covalent hydrogen bonding generated between urethane esters and tissues	Polyurethane	*In vivo* bone repair	(Lei et al., 2019)

### Nerve Regeneration

Peripheral nerve defect is a common injury and often lead to partial or complete loss of sensation or even permanent disability (Ichihara et al., 2008). Although nerves have an inherent regenerative capacity, transected nerves typically show hindered regeneration. Such damages often require surgical interventions (Soucy et al., 2018). Methods such as autografts have shown success in treating the damaged nerve, but they have limitations such as surgical incisions, donor site morbidity, and limited graft supply (Ray and Mackinnon, 2010). As an alternative, tissue scaffolds, or conduits, have been fabricated and used as bridges for damaged nerve reconnection. They have shown great promise to facilitate and guide the regrowth of neurites (Stephanie et al., 2017; Ning et al., 2019). The standard approach to connect the transected nerve with a scaffold is suturing which can mechanically fixate the artificial structure into the nerve (Barton et al., 2014). However, suturing has inadequate capacity for sealing the nerves, and also could cause secondary damages to the injured tissue and increase tension, which would lead to reduced angiogenesis, one of the key requirements for nerve regeneration (Kehoe et al., 2012; Bahm et al., 2018). Therefore, sutureless approaches, such as bioadhesives, have been developed and attracted increasing attention. They seal the sectioned nerves and bind the two tissue ends together. Among these adhesives, fibrin-based glues have been extensively used (Sameem et al., 2011; Wang W. et al., 2018). The infilled fibrin glues could further guide the growing direction of neurites with orientated microfibers, while inhibiting the encroachment of scar tissue (Liqun et al., 2018). However, fibrin glues do not fulfill the mechanical and adhesive strength requirements for neural repair under sutureless conditions due to their inherently low stiffness. Also, these glue materials pose a high risk of infection which further limits its applications (Mehall et al., 2002). Compared to fibrin, cyanoacrylate gules overcome the infection risks, but their inferior biocompatibility could lead to possible foreign body reaction and fibrosis (Wieken et al., 2003). Another hydrogel-type glue, PEG, has also been used in nerve repair (Robinson and Madison, 2016). PEG glue has the potential of incorporating bioactive molecules through covalent reaction and has short binding time under initiation of visible light. The major concern for using PEG-based glues is the low degradation rate, hence, the possibility of persisting graft years after implantation (Barton et al., 2014).

**FIGURE 3:**
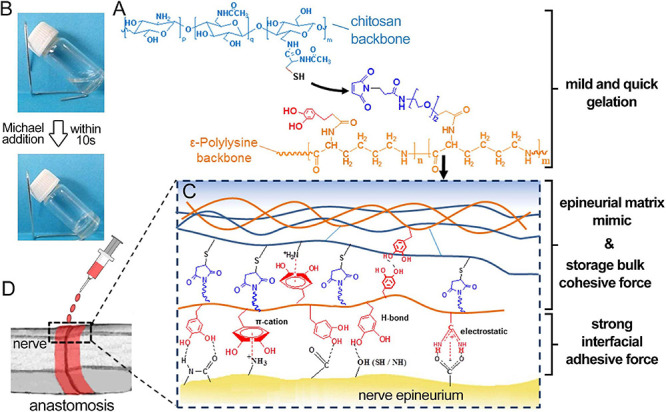
Application of adhesive tissue engineering scaffold (ATES) in nerve repair. **(A)** Mechanism of Hydrogel Formation. **(B)** Transforming from prepolymer solution to hydrogel state. **(C)** Mechanism of adhesion between the hydrogel and the nerve epineurium. **(D)** Schematic demonstrating the application of ATES *in vivo*. Reconstructed with permission from Zhou et al. (2016).

As an alternative to these conventional suturing and glue methods, ATESs can be used as an advanced approach that offers greater biocompatibility, lower secondary damage, adjustable adhesive and mechanical strength, and tunable shapes. For instance, composite scaffold systems have been made by photocrosslinking two natural polymers, gelatin-methacryloyl (gelMA), and methacryloyl-substituted tropoelastin (MeTro) (Soucy et al., 2018). The gelMA/MeTro scaffold demonstrated tunable physicochemical properties, such as degradation rate, that could be regulated to match the nerve growth rate. These hydrogels exhibited a remarkable adhesive strength to the nerve tissue, 15-fold greater than the control fibrin glue. Another example is a chitosan and catechol modified ε-polylysine (PL) based adhesive hydrogel. The adhesion ability comes from non-covalent hydrogen bonding, π-cation and ionic interactions formed between catechol and lysine groups with nerve epineurium. *In vivo* tests demonstrated the ability of re-connecting and repairing of transected nerve fiber (Figure 3; Zhou et al., 2016).

Future steps to further improve the function of ATESs in nerve regeneration include designing structures that could direct neural growth, incorporation of growth factors to promote cellular proliferation and/or function and integrating electrical stimulators or conductors. Also, the accuracy of axon reconnection with their original end-organ targets is essential because otherwise the generated nerve could be functionally compromised.

### Use of ATESs in Cartilage Repair

**FIGURE 4:**
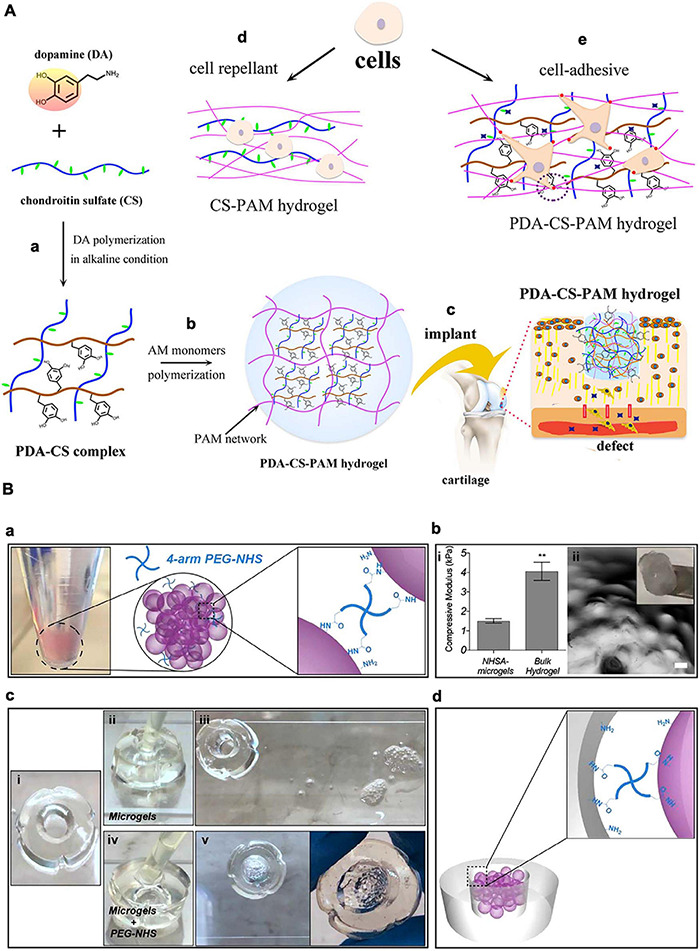
Application of adhesive tissue engineering scaffolds (ATESs) in cartilage repair. **(A)** PDA-CS-PAM adhesive scaffold to regenerate cartilage. **(a)** Mechanism of PDA-CS complex fabrication. **(b)** Mechanism of PDA-CS-PAM hydrogel formation. **(c)** Schematic demonstration of the application of adhesive scaffold *in vivo*. **(d)** Cell repellence of CS-PAM hydrogel. **(e)** Promotion of cell adhesion to the hydrogel by addition of PDA. **(B)** Adhesive microgel systems for cartilage tissue engineering. **(a)** Assembly of microspheres induced by 4-arm PEG-NHS. **(b)** Assembled NHSA-microgels: **(i)** Compressive modulus of NHSA micro and bulk hydrogels by unconfined compression test; **(ii)** NHSA microgels on a spatula and under microscope (scale bar: 100 μm). **(c)** *In vitro* testing of adhesion ability: (**i**) Hollow gelatin hydrogel; **(ii,iii)** Injection of untreated microgels into the middle of the hollow hydrogel and no adhesion after 90 min; **(iv,v)** Injection of PEG-NHS treated microgel suspension into the middle of the hollow hydrogel and adhesion after incubation. **(d)** Demonstration of adhesion mechanism between microgels and tissue. **P < 0.01. Reconstructed with permissions from Fanyi et al. (2018) and Han et al. (2018).

Cartilage regeneration is vital for mitigating osteoarthritis, orthopedic trauma, meniscus damage, and other degenerative diseases (Kim et al., 2018). The lack of vasculature and nerve system, together with limited migration ability of chondrocytes, impair cartilage self-regeneration capacity. When left untreated, these conditions lead to loss of mobility and advance into chronic diseases (Fanyi et al., 2018). One effective therapy could be scaffolds that are biocompatible and adhere to the entire damaged tissue, and can maintain encapsulated cells, promote chondrocyte proliferation, and activate glycosaminoglycan (GAG) and collagen secretion. Traditional scaffold grafting methods are suturing and glue. In the case of cartilage, suturing can cause loss of chondrocytes and proteoglycans, induced by insertion of the suturing needle, fissures in the wall of the suture channels, and propagation of exiting cracks under mechanical forces acting on the joint (Hunziker and Stähli, 2008). Glues could also cause various complication that were described in previous sections. For maxillofacial cartilage specifically, material fixation methods should bring minimal physical damage to nerves, have low infection rate, and be non-toxic considering their short distance to the brain. Therefore, conventional suture and glue methods are often challenging for the fixation of materials onto the maxillofacial cartilage. ATESs can solve this problem by offering great biocompatibility, minimally invasive delivery approach through syringes, and tunable and short adhesion time post-delivery (Salzlechner et al., 2020). For instance, a hyaluronic acid (HA) hydrogel, modified with both MA and Dopa groups, was applied under aqueous conditions, demonstrating a rapid gelation using a standard surgical light and an adequate adhesion to the muscle tissue (Salzlechner et al., 2020). The catechol functional groups in dopamine can bind to organic and inorganic substrates through covalent and non-covalent interactions and are frequently used in bio-adhesive materials as dopamine can be readily incorporated into the backbone of polymers. Another type of catechol based ATESs is fabricated by polydopamine-chondroitin complex and polyacrylamide, with the adhesion property coming from the non-covalent interactions brought by catechol (Han et al., 2018). The hydrogel supports proliferation and gene expression of bone marrow stem cells and chondrocytes *in vitro.* Further, *in vivo* experiments demonstrated the ability of the adhesive hydrogel to repair cartilage defects (Figure 4A).

Injectable hydrogels also offer a minimally invasive nature and could fit into complex and irregular geometries of the degenerated cartilage (Liu M. et al., 2017). Hydrogel scaffolds could provide protection against shear forces during injection and increase retention rate of cells as well as support cell migration, proliferation, and function (Liu M. et al., 2017; Li et al., 2019). The intrinsic adhesive ability of injectable hydrogels is essential for fixation of deposited biomaterial onto the damaged cartilage. Polymers such as gelatin, HA, and alginate could be used to develop injectable hydrogels with intrinsic adhesive properties. Functional groups in these polymers are used for the incorporation of crosslinkable moieties that can form covalent bonding with chemical groups, such as primary amines, on the cartilage tissue. For instance, oxidation can create functional groups such as aldehydes in alginate polymers. An adhesive injectable hydrogel scaffold composed of oxidized alginate, gelatin, and borax (as crosslinker) has been used to encapsulate chondrocytes (Balakrishnan et al., 2014). Alginate induced chondrocytes re-differentiation and gelatin promoted hydrogel-chondrocyte interaction. Aldehyde groups in oxidized alginate reacted with amine groups in gelatin and the surrounding tissue to elicit crosslinking of hydrogel and adhesion to the cartilage (Balakrishnan et al., 2014). In more recent works, an catechol modified chondroitin sulfate based adhesive hydrogel was fabricated for cartilage regeneration (Shin et al., 2021). Further, an injectable catechol group modified poly(2-alkyl-2-oxazoline) and fibrinogen based material was developed to treat cartilage defects (Berberich et al., 2019). Results from these studies suggested that injectable adhesive hydrogels solutions may provide an optimal solution for cartilage (and other) tissue repair due to their high tunability.

In another study, tyramine (TA) conjugated HA (TA-HA) combined with gelatin was used to form adhesive injectable hydrogel by tyrosinase (TYR)-mediated crosslinking and adhesion. HA is one of the major components of cartilage ECM and could help with cell proliferation, migration, and tissue regeneration. TA can be oxidized by TYR to generate quinone groups. These groups covalently bond with other phenolic moieties or amine, thiol, and imidazole groups in gelatin, for crosslinking, and with groups on tissue for adhesion (Kim et al., 2018). These adhesive injectable hydrogels encapsulated with cells can facilitate tissue regeneration through minimally invasive procedures.

To further facilitate nutrient and waste exchange within the ATES structure, microgel spheres have been also adapted as scaffold systems for articular cartilage repair (Figure 4B; Fanyi et al., 2018). To achieve rapid bonding between microgels for assembling into higher order structures, and also the adhesion between microgels and the surrounding tissue, 4-arm poly (ethylene glycol)-Nhydroxysuccinimide (NHS) has been used as a crosslinker (Fanyi et al., 2018). NIH forms covalent bonds with primary amines on gelatin based microgels and with the protein-rich cartilage tissue. Human bone marrow derived mesenchymal stem cells (hBMSCs) were encapsulated in these microgel assemblies exhibited significantly increased chondrogenic gene expression.

In summary, ATESs can aid cartilage regenerative processes by offering a minimally invasive delivery, a simplified surgical grafting operation, flexibility to treat small or complex defects, steady fixation without secondary damages or cytotoxicity, protection and retention of cells, and support of cellular proliferation and differentiation. In addition to these benefits, an ideal scaffold for cartilage repair should also demonstrate adequate tolerance to certain levels of forces and maintain effective adhesive strength under dynamic movements. These requirements must be addressed in the future endeavors on biomaterial selection and development.

### Corneal Regeneration Using ATESs

If left untreated, corneal injuries and infections might lead to eye shape deformations and even vision loss. Tissue grafting and corneal transplantation are standard treatments for corneal stromal defects (Yorston and Garg, 2009). Tissue grafting is limited by the need for donor tissues, special equipment, and advanced skills, as well as potential transplant complications and rejection. Corneal transplantation has drawbacks brought by donor tissue shortage and high expense of transplantation surgery. Also, if suturing is applied, the process not only requires high skill levels from the surgery team and a relatively long time for operation, but can also cause multiple complications including inflammation, astigmatism, suture breakage, secondary neovascularization, microbial infection, as well as the lack of control of disease recurrence (Chan and Boisjoly, 2004; Bhatia, 2006; Grinstaff, 2007; Romano et al., 2016; Santiago et al., 2019). Ocular adhesives used as an alternative for the above treatments typically consist of synthetic materials, such as cyanoacrylate-based, PEG-based, and dendrimer-based adhesives, and naturally derived materials, such as protein-based and polysaccharide-based adhesives (Park et al., 2011; Koivusalo et al., 2019; Santiago et al., 2019). In particular, cyanoacrylate-based glues, PEG-based adhesives, and fibrin glues have been most commonly used in treatments for various ocular conditions (Santiago et al., 2019).

Cyanoacrylates are one of the earliest ocular adhesive solutions used. However, their cytotoxicity, rough texture, poor biodegradability and bioabsorbability, inflexibility after solidification, and lack of transparency impose major limitations on their application in clinical treatments (Ciapetti et al., 1994; Kaufman et al., 2003; Chan and Boisjoly, 2004; Bhatia, 2006; Chen et al., 2007; Park et al., 2011). An FDA-approved PEG-based adhesive for ocular repair, ReSure^®^, has already been used in cataract surgery and laser-assisted *in situ* keratomileusis (LASIK) surgery (Masket et al., 2014; Ramsook and Hersh, 2015; Tong et al., 2018). But this hydrogel adhesive requires rapid operation, has limited stability, cannot seal large leaky incisions, or fill in stromal defects (Park et al., 2011). The drawbacks of fibrin glue mainly lie in its poor adhesion ability to wet surfaces, difficulty to control product quality, and potential risks of viral contamination and immunological problems (Shirzaei Sani et al., 2019).

**FIGURE 5:**
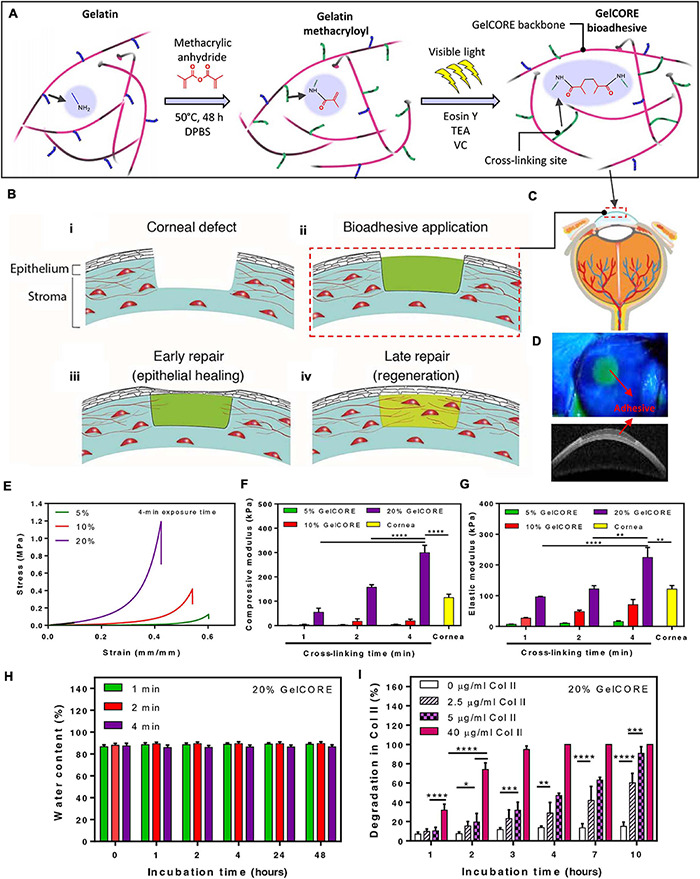
Application of adhesive tissue engineering scaffolds (ATESs) in cornea repair. **(A)** Mechanism of hydrogel formation. **(B)** Application of ATES: **(i)** Corneal defect; **(ii)** Scaffold application; **(iii)** Epithelial healing; **(iv)** Regeneration. **(C)** Injection of prepolymer into injured cornea. **(D)** Demonstration of GelCORE hydrogel. **(E–G)** Compressive stress-strain curve **(E)**, compressive moduli **(F)**, and elastic moduli **(G)** for GelCORE hydrogels at varied concentration and crosslinking time. **(H)** Water content of GelCORE hydrogel after different crosslinking times at 37°C. **(I)** GelCORE degradation in collagenase type II at 37°C. *P < 0.05, **P < 0.01, ***P < 0.001, and ****P < 0.0001. Reconstructed with permission from Shirzaei Sani et al. (2019).

For an optimal alternative adhesion strategy for corneal tissue grafting or transplantation, the following biomaterial requirements must be fulfilled: long time stability onto the tissue, transparency, biocompatibility and biodegradability, appropriate stiffness, support of cell growth, simplified application procedure, and the ability to elicit tissue regeneration (Ahearne et al., 2020). ATESs are considered a proper candidate, as they can support tissue regeneration, have designable degradation rates and stiffness, and avoid suturing and glues, which is essential when it comes to vision recovery (Figure 5). Adhesive scaffolds are also able to fill larger stromal defects and help reduce surgery difficulties with intrinsic bonding ability. For instance, a modified photo-crosslinkable gelatin material, called GelCORE (gel for corneal regeneration), has been shown to adhere to the cornea tissue within a short-time exposure to visible light (Figure 5; Shirzaei Sani et al., 2019). While an adhesion strength higher than the commercially available adhesives was obtained, this ATES also maintained transparency. GelCORE was compatible with corneal cells and promoted cell integration. It effectively sealed corneal defect and promoted re-epithelialization (Shirzaei Sani et al., 2019). Another ATES product was based on modified HA. Two components, aldehyde modified HA and carbodihydrazide and dopamine modified HA, were mixed to fabricate a transparent hydrogel (Koivusalo et al., 2019). Aldehyde groups formed covalent bonding with the surrounding tissue, while dopamine groups augmented the adhesion strength and promoted human adipose-derived stem cell (hASC) culture by facilitating the conjugation of cell-adhesive proteins to the hydrogel surface. One novelty of this approach was the co-encapsulation of two cell types, hASCs and human embryonic stem cells (hESCs) into the ATES, with hASCs buried within the hydrogel to elicit regeneration of corneal stroma and hESCs on the surface of for regeneration of epithelium (Koivusalo et al., 2019).

For corneal regeneration, aside from basic requirements such as biocompatibility and biodegradability, transparency and appropriate stiffness for patient comfort are key factors for an optimal scaffold design. ATESs can help avoid complications caused by suturing, such as astigmatism and extensive neovascularization, as well as circumvent the inability of glue products in filling the stromal defects. Transparent ATESs with adjustable mechanical strength that could adhere to the tissue for long periods of time offer great promise as a desirable tissue engineering device for ocular regeneration.

### ATESs in Skin Regeneration

**FIGURE 6:**
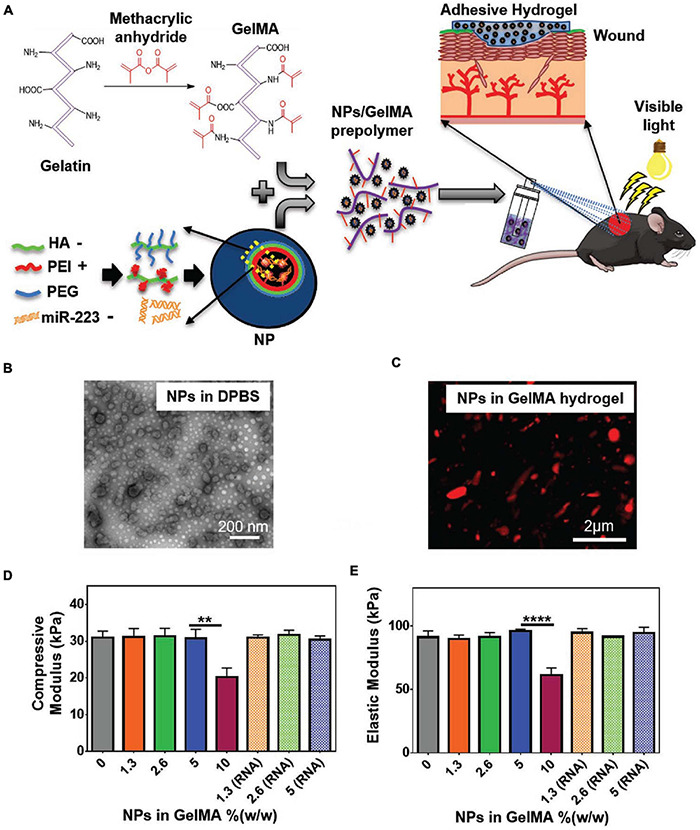
Application of adhesive tissue engineering scaffolds (ATESs) in skin tissue repair. **(A)** GelMA hydrogel formation and application to skin wounds. **(B)** Representative TEM image of HA/miR-223^∗^ NPs with ratio of 325:1 (w/w) in DPBS. **(C)** Representative confocal image of Cy5.5-labeled (red) NPs in hydrogel. **(D)** Elastic modulus of hydrogels containing different NP concentrations. **(E)** Compressive modulus of hydrogels with different NP concentrations. **P < 0.01 and ****P < 0.0001. Reconstructed with permission from Saleh et al. (2019).

While treatments for acute skin wounds can be effective, chronic wounds can be difficult to fully treat (Subhamoy and Baker, 2016). Further, diabetes, severe burning, or other severe conditions may obstruct the natural healing process of the skin, highlighting the need for enhanced clinical interventions (Chouhan et al., 2019). Among alternative strategies, cultured epithelial autograft (CEA) sheets, skin grafts, skin substitutes, wound dressings, and injectable hydrogels are commonly used to facilitate wound healing (Vig et al., 2017). CEA sheets limitations include relatively long preparation time and poor function in full thickness wounds. Skin grafts are invasive and may cause complications such as scarring and infection (Tottoli et al., 2020). Skin substitutes are tissue engineered products that are designed to replace or mimic the form and function of the skin (Krishna et al., 2014). Wound dressings work as a barrier for maintaining moisture and keeping out bacterial infections (Zoe et al., 2019). Engineered scaffolds are commonly used as skin substitutes and wound dressings and can facilitate healing process through providing a reservoir of cells and growth factors to mediate angiogenesis, inflammation, antibacterial properties (Figure 6). At the same time, these ATESs can regulate cell infiltration, proliferation, and replacement of the lost tissue (Boateng et al., 2008). Adhesive properties of tissue engineering scaffolds can have several benefits for their application in wound healing: (1) these constructs are able to conform to uneven, curved, or folded surfaces of complex skin wounds or wounds near joints; (2) can reduce the risk of wound exposure to bacterial invasion; (3) avoid the use of sutures and staples that can cause secondary damages to the tissue; and (4) the adhesive *in situ* forming hydrogels can be applied to longitudinal wounds to protect them from the external environment (Saleh et al., 2019; Hou et al., 2020b). Also, adhesion ability combined with *in situ* gelling ability can be used to fabricate injectable hydrogels that can fill wounds with irregular shapes and provide a customized coverage (Subhamoy and Baker, 2016; Duy et al., 2018).

Patch shaped polyurethane-poly(acrylamide) (PU-PAAm) hydrogels can be UV cured to demonstrate tissue adhesion functionality that is introduced by electrostatic interactions between hydrogel and the skin (Hou et al., 2020b). Further, the interpenetration of PU and PAAm gives stretchability and ductility to the hydrogel. The adhesion property, along with the flexibility, allow this ATES to fit into complex wounds and prevent bacterial invasion. The hydrogel shows remarkable skin regenerative capacity and easy removal (Hou et al., 2020b). Compared with control group, the hydrogel treatment groups showed decreased inflammatory cells infiltration and enhanced vascularization and epithelialization. UV-crosslinkable gelMA-based hydrogels that are supportive of cell attachment, infiltration, and proliferation, can be also used to mediate wound re-epithelialization and healing (Figure 6; Saleh et al., 2019). The adhesion strength is brought by mechanical interlocking between gelMA and the native tissue, as well as covalent bonding triggered by radicals generated during photo-crosslinking.

Injectable adhesive hydrogels can also fill irregular shape wounds by *in situ* gelation and adhesion that could be used to heal longitudinal wounds. An example is a multiblock copolymer, comprised of PEG and pH- and temperature-sensitive poly(sulfamethazine ester urethane) (PSMEU), which can change from sols into stable gel by transitioning from *in vitro* (8.5, 23°C) to *in vivo* conditions (7.4, 37°C) (Duy et al., 2018). The adhesion ability came from the presence of urethane esters that interact with the tissue. Quantitative measurement of wound closure rate, breaking strength, and collagen content suggested that the adhesive hydrogel effectively homed the cells, facilitated cell migration, and provided a suitable environment for neo-tissue formation (Duy et al., 2018). Another example is poly (ε-caprolactone-*co*-lactide)-*b*-poly-(ethylene glycol)-*b*-poly (ε-caprolactone-*co*-lactide) (PCLA-*b*-PEG-*b*-PCLA, called in short PCLA) which is a biodegradable temperature sensitive polymer. PCLA/gelatin hydrogels with cell affinity and porous structure are used to seal the wounds and promote wound healing (Li et al., 2020). The adhesive ability can come from the interfacial hydrogen bonding between hydrogels and skin tissue. The presence of gelatin could improve the adhesion strength due to the ionic interactions between free amine groups on the gelatin chains and the skin tissue. In one study, the PCLA/gelatin hydrogel treated groups showed well organized collagen fiber and complete re-epithelialization after 7 days in a simple liner wound model with a 1 cm cut (Turabee et al., 2019). Further, treatment of a full thickness wound model with a 1 cm × 1 cm excisional wound showed granular tissue formation, dermis deposition, and enhanced collagen remodeling, suggesting that the scaffold provides a suitable environment for neovascularization and tissue regeneration (Turabee et al., 2019). More recently, a polydopamine-sodium alginate–polyacrylamide (PDA–SA–PAM)-based hydrogel with multi-functions was developed for skin tissue engineering (Suneetha et al., 2019). These hydrogels can be used as drug delivery systems for targeted and sustained release, hence, reducing systemic drug toxicity.

In sum, the application of ATESs in the fields of wound healing and skin tissue engineering has shown great promise. Future works could focus on developing an optimal adhesive scaffold with the following properties: (1) ability to adsorb wound exudates while maintaining moisture; (2) ability to protect the wound from the external environment, bacteria, and other pathogens; (3) flexibility and adaptability to complex wound shapes that enable complete and customized coverage; (4) applicability to all healing phases; and (5) basic functions such as biocompatibility, biodegradability, low cytotoxicity, and oxygen permeability, cost effectiveness, availability, and easy storage and application (Rezaie et al., 2019).

### The Use of ATESs in Cardiac Tissue Repair

Adult cardiomyocytes have limited capacity for replication, which leads to the requirement of effective therapies to help regenerate damaged heart tissue (Ruvinov and Cohen, 2013; Doppler et al., 2017). A variety of tissue engineering therapies have been investigated for myocardial repair, including cell-based and scaffold-based approaches. Each strategy has its own challenges. The limitations of cell injections include the low cell survival and retention rate, limited interaction between the transplanted cells and the host tissue, and the possibility of inducing or exacerbating arrhythmias post injection (Soler-Botija et al., 2012; Huang et al., 2018, 2019). Injectable hydrogels might cause secondary damages and hemorrhage to the already weakened heart, and also limit the amount of therapeutics and cells that can be delivered to the tissue due to the hydrogel-induced pressure (Shin et al., 2019). Cell sheets face the challenge of electromechanical isolation from the native myocardium, as well as vascularization resistance when the construct contains four or more cell layers (Zhang and Jianyi, 2015). Cardiac patch devices are an alternative for the treatment of cardiovascular tissues after severe injuries (Serpooshan and Ruiz-Lozano, 2014; Serpooshan et al., 2014; Mei and Cheng, 2020). These engineered scaffolds can act as a depository of regenerative factors and a matrix to aid targeted therapeutic delivery and sustained release (Tomov et al., 2019). In comparison to other types of treatments, patch devices can offer the following benefits: (1) pre-designed structure that could be patient-specific, incorporate vasculature and contain patterned cells according to desired function (Noor et al., 2019); (2) full coverage of the entire damaged area (and the area at risk) that is specifically important for drug delivery and mechanical support (Ravichandran et al., 2015); (3) the ability of adhesive scaffolds to reduce secondary damages to the injured tissue, which is typically associated with gluing or intramyocardial injection methods. Using adhesive and glue materials is typically associated with challenges such as the inhibition of cell migration from the patch to damaged tissue, inadequate stiffness, cytotoxicity and exothermic reaction by polymerization of cyanoacrylates, complications related to viral infections, and low adhesion ability of fibrin sealants (Shin et al., 2019).

A ready-to-use tissue-adhesive catechol or pyrogallol modified HA patch has been developed and used for cardiac cell and drug delivery (Shin et al., 2019). The phenolic HA patches were lyophilized before use and simply applied by placing the hydrogel onto the cardiac tissue surface. Tissue adhesion and polymer crosslinking were initiated by oxidation of catechol or pyrogallol through spraying oxidizing solution (4.5 mg/mL of NaIO_4_). Rat BMSCs were transplanted by seeding onto the adhered patches. The lyophilized patch soaked up water molecules and formed a hydrogel after adhesion. The lyophilization step significantly enhanced the adhesion ability of the hydrogel. Application of the adhesive patch prevented LV dilatation and cardiac hypertrophy, and improved angiogenesis in a rat model of myocardial infarction (MI) (Shin et al., 2019).

**FIGURE 7:**
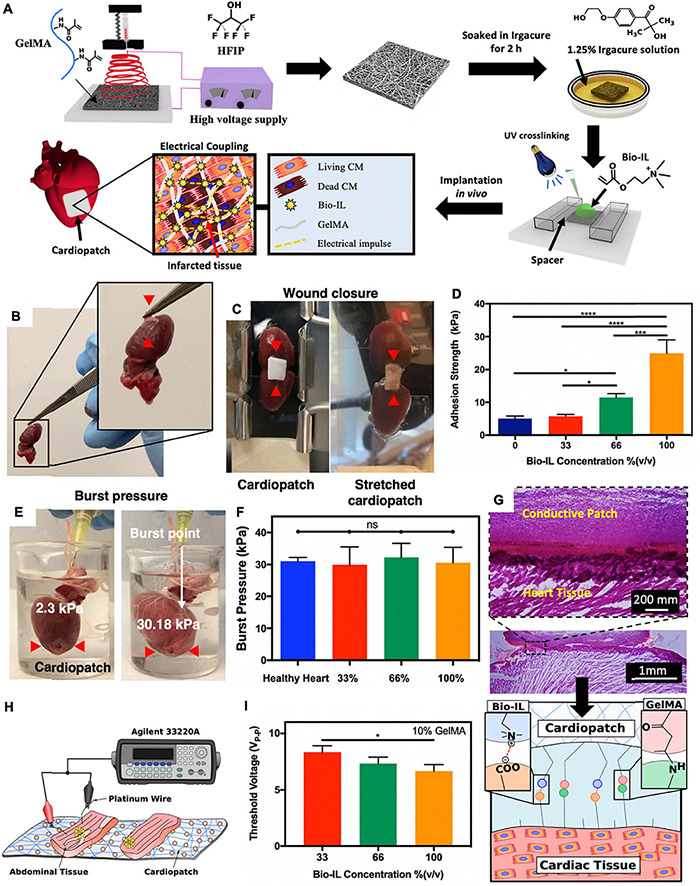
Application of adhesive tissue engineering scaffolds (ATESs) in cardiac tissue repair. **(A)** Fabrication of electrospun cardiopatches, soaking in Irgacure solution, addition of Bio-IL, followed by UV crosslinking for 5 min. **(B)** GelMA/Bio-IL cardiopatch photo-crosslinked on explanted rat heart, demonstrating adequate adhesion (red arrows) to the heart tissue. **(C)** Wound closure test to test the adhesion strength of cardiopatches on the explanted rat heart (as substrate). **(D)** Quantification of the patch adhesion strength, consisting of 10% (w/v) gelMA and at varying concentrations of Bio-IL. **(E)** Images of gelMA/Bio-IL cardiopatch with 10% gelMA and 66% Bio-IL, crosslinked onto the defect site of explanted rat heart, to measure the burst pressure. **(F)** Quantification of the burst pressure. **(G)** H&E staining of patch-tissue interface, demonstrating a strong bonding of the hydrogel to the murine myocardium. **(H,I)** *Ex vivo* analysis of the threshold voltage of gelMA/Bio-IL cardiopatches at varying Bio-IL concentrations. *P < 0.05, ****P < 0.001 and ****P < 0.0001. Reconstructed with permission from Walker et al. (2019).

Electrospun fibrillar patches can also serve as ATESs for cardiac tissue regeneration. The benefits of using fibrillar scaffolds include high surface area to volume ratios, defined spatial density, 3D anisotropic organization, and recapitulation of the fibrillar topography of the native myocardium (Kim and Cho, 2016; Streeter et al., 2019). For instance, a gelMA-based fibrillar patch was developed that could adhere to the heart tissue via photo-crosslinking, during which, MA groups of gelMA formed covalent bonds with amine groups of the tissue (Figure 7; Walker et al., 2019). To restore electrical conductivity at the site of MI, a choline-based bio-ionic liquid (Bio-IL) was covalently bound to the gelMA patch during photocrosslinking. The Bio-IL contributed to the adhesion ability through electrostatic interactions between its positive charges and negative charges of carboxyl groups in the cardiac tissue. After *in vivo* implantation, both gelMA and gelMA/Bio-IL scaffolds demonstrated tissue ingrowth, suggesting that they could both provide a cell-supportive microenvironment to reduce adverse cardiac remodeling post MI (Figure 7; Walker et al., 2019).

Fibrillar scaffolds can also adhere to the tissue through near IR irradiation, which results in hear generation and partial and local melting (denaturing) of the scaffold polymer to evoke binding and adhesion. An example is an electrospun albumin-based scaffold, encapsulating gold nanorods (AuNRs) (Malki et al., 2018). AuNRs absorb near IR radiation and generate heat, while simultaneously can help increasing electrical conductivity of the hydrogel matrix grafted onto the MI tissue. To inhibit potential damage of the localized heat to tissue, the patch was irradiated to attach to the heart tissue only on the peripheral areas, resulting in a strong fixation of the construct for the entire duration of assays (Malki et al., 2018).

In summary, adhesion to the heart tissue is a difficult task due to the presence of a high density of blood vessels and the highly dynamic forces. In addition to adequate adhesive strength, several other essential factors must be considered in fabrication of optimal cardiac ATES. These include electromechanical coupling of the patch with the host tissue, proper cell type, density, and distribution within the scaffold, and sufficient mass transport properties. Efforts should be made to integrate intrinsic adhesive properties, with other key requirements specific to cardiac tissue grafts. 3D bioprinting technologies that have already shown great promise in customization of cardiac patch structure and function (Hu et al., 2017; Serpooshan et al., 2018), could be an important tool in the design and development of cardiac ATES systems. For instance, an *in vivo* printed gelMA based adhesive scaffold was developed and used for skeletal muscle tissue repair (Quint et al., 2021).

### ATES Solutions for Bone Repair

Regeneration of bone tissue when its remodeling capacity cannot compensate the tissue destruction remains a challenge in clinical practice (Yin et al., 2019; Bai et al., 2020). Treatment of such bone defects typically requires filling by autologous or allogenic grafts, as well as stabilization by screws, cages, or rods (Duarte et al., 2020). Limitations of autografts include excessive pain, donor site morbidity, cost, and limited supply. Allografts, with abundant source, are challenged by the uncertainty of compatibility and suboptimal osteoinductivity which may result in delayed or incomplete bone regeneration, immunogenic reactions, risk of infection, and possible disease transmission (Stevens et al., 2010; Gibbs et al., 2016). Complications associated with metal tools such as screws are potential over-tightening and bone stripping, fixture dislocation, fractures from holes, bone resorption by stress shielding, foreign-body reactions, growth disturbance, and the possible surgery for their removal (Schreader et al., 2012; Lei et al., 2019; Bai et al., 2020). Traditional bone adhesives, as an alternative, help with the spread of force over the whole contact area and thus minimize stress shielding effects. However, commonly used poly (methyl methacrylate) (PMMA) bone cement has low biocompatibility, no intrinsic adhesion ability to the bone, toxic monomers, poor bioresorbability, and possible thermal damage during polymerization. Fibrin glue is limited by its low mechanical properties and risk of inducing allergies. Calcium phosphate bone cements (CPCs) often lack proper mechanical properties and adhesive ability (Lei et al., 2019; Bai et al., 2020; Duarte et al., 2020). Advanced tissue engineering scaffolds with adhesive properties in wet environment, can replace autologous or allogenic bone grafts by offering sufficient biocompatibility and biodegradability. Further, ATESs will serve as a reservoir for cells and growth factors, as well as a suitable microenvironment that directs cell proliferation and differentiation toward bone regeneration (Leberfinger et al., 2017; Hasani-Sadrabadi et al., 2020).

**FIGURE 8:**
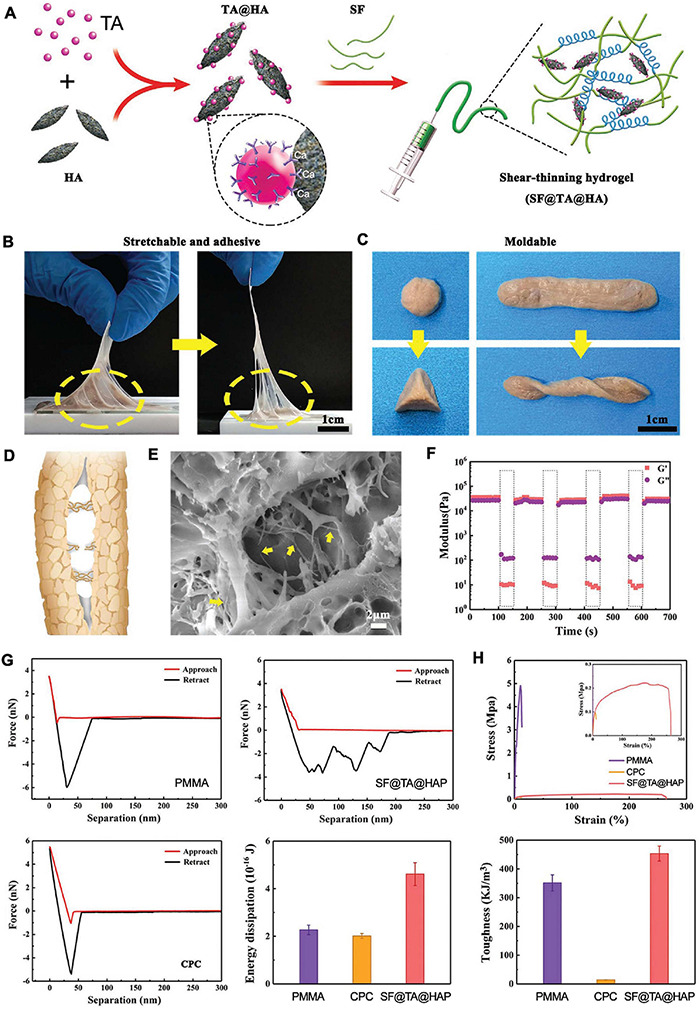
Application of adhesive tissue engineering scaffolds (ATESs) in bone tissue engineering. **(A)** Demonstration of SF@TA@HAP hydrogel formation. **(B)** Demonstration of adhesion and stretchability of SF@TA@HAP scaffold. **(C)** Demonstration of the flexibility and malleability of the hydrogel. **(D)** Glue filaments in the bone structure, connecting mineralized collagen fibrils. **(E)** Representative SEM image of the filaments in the SF@TA@HAP hydrogel. **(F)** Modulus of SF@TA@HAP hydrogel under repeated application of 100 and 0.1% strain. **(G)** AFM mechanical testing of SF@TA@HA, PMMA, and CPC. Bar graphs show the quantified values of dissipated energy during the separation step. **(H)** Results of mechanical testing of SF@TA@HA, PMMA, and CPC samples. Bar graphs show the quantified toughness. Reconstructed with permission from Bai et al. (2020).

An inorganic–organic hybrid scaffold consisting of tannic acid (TA), silk fibroin (SF), and hydroxyapatite (HAP) has been developed as an ATES for bone regeneration (Figure 8; Bai et al., 2020). This SF@TA@HAP system was inspired by the human bone, where inorganic nanoparticles are glued into organic collagen by proteins and proteoglycans. The bone hierarchical organization uses calcium-mediated sacrificial bonds for energy dissipation, ensuring high mechanical strength and healing properties. In the case of this bone-mimetic hybrid hydrogel, tannic acid acts as the glue to combine inorganic HAP and organic SF to form a scaffold with robust water-resistant structure (Bai et al., 2020). The adhesion ability comes from Ca^2+^ – phenolic bonds and other nucleophile-phenolic non-covalent interactions between the scaffold and the collagen proteins and HAP of bone tissue. The scaffold, embedded with bone morphogenetic protein-2 (BMP-2), guided MSCs toward osteogenic differentiation and mineralization *in vitro*. Further, application of the ATES in a rat femoral defect model resulted in enhanced bone regeneration bridging across the defect (Bai et al., 2020). Another ATES device was developed using dopamine–modified alginate and used to deliver cells and guide mineralization (Hasani-Sadrabadi et al., 2020). Moreover, a porous room-temperature-cured foam-like adhesive scaffold, based on polyurethane, was tested *in vivo*, demonstrating extensive cellular infiltration and newly generated bone, forming a connected structure after 24 weeks of osteotomy (Lei et al., 2019). More recently, an injectable alginate based adhesive hydrogel, laden with mesenchymal stem cells, was developed for craniofacial tissue engineering (Hasani-Sadrabadi et al., 2020).

In summary, ATESs could offer a highly attractive alternative therapy to substitute autologous or allogenic bone grafts, as well as serving as a highly tunable bone adhesive, replacing screws and other metallic devices for fracture stabilization. An ideal adhesive scaffold in the future should be able to adhere under wet conditions, while exhibiting adequate mechanical strength, especially compressive strength.

## Current Challenges and Future Prospective

Design and development of ATESs have attracted increasing attention in recent years. Recent works have successfully created scaffold systems that fulfill the basic requirements (elastic modulus, adhesion strength and mechanism, degradation rate, and biocompatibility) for diverse tissue engineering applications. However, efforts are still needed to enable and enhance clinical translation of these products in the future. Current adhesive scaffolds are mostly made through traditional scaffold fabrication methods, such as *in situ* gelation from liquid pre-gel. Integration of advanced scaffold fabrication methods, such as 3D printing and bioprinting, could be considered for enhanced cell-biomaterial arrangement, vessel incorporation, and personalized designs. Maintaining proper adhesion properties under wet conditions is still a challenge. New mechanisms that help tolerating such harsh conditions should be investigated for successful clinical translation. Also, methods for less invasive delivery of adhesive scaffolds should be adopted for each specific tissue and organ. Since additional fixation is not required for adhesive scaffolds, delivery through minimally invasive conduits (e.g., catheters) could be considered.

Effective application of ATES devices in different tissue engineering applications currently face many challenges. ATESs for all different types of tissues face challenges such as wet environment where water molecules form a boundary and mask functional groups, low pH, oxidative environment, high immune response under trauma and post-surgery. For the nerve scaffolds, significant tensile stresses are another challenge. In cartilage and bone, dynamic compressive stresses would complicate the adhesion requirements. In the case of corneal tissue engineering, the ATES must also exhibit adequate transparency and patient comfort. Finally, for the cardiac scaffolds, dynamic loading exerted by the beating heart and the lack of reservoir which requires quick adhesion, are some of the key limitations.

Future research on adhesive scaffold devices could focus on two specific directions: (1) advanced adhesion properties that can maintain the secured device attachment under harsh conditions such as bleeding, dynamic (pulsatile) loading such as beating heart, or in the presence of strong immune responses; (2) Incorporation of adhesive hydrogel (biomaterial) technologies into advanced tissue biomanufacturing techniques. In particular, adhesive scaffold devices can be 3D bioprinted to provide a more targeted and personalized structure, while incorporating more functional features such as perfusable vascular networks and heterogeneous cellular populations that more closely mimic the native human tissue.

## Conclusion

Adhesive tissue engineering scaffolds are advanced medical treatments to replace traditional fixation strategies such as sutures or bio-glues to circumvent their drawbacks such as morbidity, toxicity, potential allergies, and operation inconveniences especially for treating complex tissue defects. Adhesive scaffolds can help with more effective cell migration and engraftment between implanted construct and the host tissue. They can also significantly reduce operation trauma and pain for patients by providing minimally invasive delivery (e.g., injection) and immediate fixation. There are substantial differences between ATESs and adhesive products. Adhesives are used for holding tissues together, while ATESs are scaffolds with *intrinsic* adhesion properties for tissue regeneration. These two functions could be held at the same time, but many adhesives lack the ability for tissue regeneration due to their low biocompatibility, insufficient mechanical properties, and improper degradation rate. Future works in ATES will focus on improving the under-water adhesion properties and simultaneously improving mechanical properties, flexibility, and other functions that are specific to each tissue and organ. For instance, scaffolding biomaterials with enhanced optical properties will be needed for corneal regeneration. Adhesion ability under dynamic forces and adequate electrical conductivity will be required to manufacture cardiac ATES devices. Furthermore, for more effective development of adhesive scaffolds, standardized testing procedures should be defined for measuring the strength of adhesion to specific tissues and organs. More insight into the adhesion mechanisms between the scaffold and tissue surfaces would be of great significance. The successful development of functional ATES products, with potential for clinical translation, could help significantly reduce patients’ pain and morbidity and therefore, is expected to draw increasing attention in the coming years.

## Author Contributions

SC, CG, LN, LJ, LP, GK, and MT contributed to writing different sections and subsections of the work, and designed and generated the figures and tables. SC led the writing tasks. VS conceived the concept, designed the overall article structure, and edited the manuscript. All authors contributed to the article and approved the submitted version.

## Conflict of Interest

The authors declare that the research was conducted in the absence of any commercial or financial relationships that could be construed as a potential conflict of interest.
